# Weighing the evidence: obesity, metabolic syndrome, and the risk of chronic kidney disease

**DOI:** 10.1186/s12882-015-0137-y

**Published:** 2015-08-07

**Authors:** Ezra Gabbay, Itzchak Slotki, Linda Shavit

**Affiliations:** Adult Nephrology Unit, Shaare Zedek Medical Center, PO Box 3235, Jerusalem, 91031 Israel

## Abstract

Evaluating effect of obesity per se and the metabolic syndrome as a whole on the risk of developing chronic kidney disease (CKD) is key factor in developing a comprehensive public health approach to reduce morbidity and healthcare resource consumption. While there is considerable evidence to support increased risk of CKD in obese individuals and those with the metabolic syndrome, this relationship may be influenced by several factors. These include confounding variables, anthropometric measures, the end-point studied (e.g. development of early stage CKD, progression to end-stage renal disease or mortality), and the complex interrelationship between the various components of the metabolic syndrome. The study by Cao et al. in the current issue of *BMC nephrology* examines the impact of obesity on CKD risk in people with and without co-existing metabolic syndrome. The findings of this large, prospective study illustrate a clear correlation between increased body mass index (BMI) and risk of CKD regardless of whether or not there is co-existing metabolic syndrome. While the presence of the metabolic syndrome confers some additional risk of CKD in overweight and obese individuals, its effect is relatively modest and accounts for only 26 % of the risk associated with increased BMI. We discuss the complex epidemiological and methodological context in which these important findings should be understood, and their implications for public health and for individual patients and healthcare practitioners.

## Background

Identifying individuals at increased risk for chronic kidney disease (CKD) is an important, yet difficult undertaking. Its importance is related both to the high prevalence of CKD [1], and to the profound effect that its progression to end stage renal disease (ESRD) has on mortality, morbidity, quality of life and healthcare resource consumption [2, 3]. Establishing the risk factors and markers for CKD is key in developing a comprehensive public health approach that may include screening, patient education, life-style modifications, pharmacotherapy and other interventions to decrease CKD and subsequent ESRD [4]. The difficulties in achieving this goal, however, are formidable. CKD is a highly heterogeneous condition. Most people with CKD have mild, asymptomatic, reductions in glomerular filtration rate (GFR), or mild proteinuria, while a minority of patients at the other end of the CKD spectrum are afflicted with ESRD, and suffer its heavy toll on their health and well-being [4]. Therefore, the meaning of an association between a given variable and CKD as a whole is often unclear. For some conditions associated with CKD, such as hypertension, distinguishing cause from effect is often problematic. Finally, the complex, often confounding, interrelationship and overlap among the many risk factors that have been implicated as contributing to the development of CKD can make it quite hard to isolate independent risk factors [5]. The case of metabolic syndrome and its components, obesity, hyperglycemia, dyslipidemia, and hypertension, illustrates the murkiness in which the epidemiological research of CKD risk must navigate.

Obesity and overweight may appear as straightforward, easily studied, potential risk factors for CKD. Indeed, compelling evidence has been found for an independent association between BMI and CKD risk in healthy men [6], and hypertensive adults [7]. One very large study of more than 320,000 patients who had undergone screening health check-ups between 1964 and 1985 found that the long term risk of ESRD was strongly and independently associated with being overweight or obese, with an odds ratio as high as 7 in extreme obesity (BMI>=40) [8]. Other studies, however, found that the apparent association of BMI with stage 3 CKD, was no longer significant after adjustment for other cardiovascular risk factors [5]. A study of patients who already had stage 3–5 CKD, showed no association between BMI and reduction in eGFR [9]. Furthermore, the location of body fat, by anthropometric measures such as waist to hip ratio (WHR) was found in some studies to be more important than BMI per se in predicting CKD and mortality [10], whereas others found waist circumference and BMI (but not WHR) to be useful predictors of CKD risk [11]. In terms of predicting ESRD and mortality in CKD patients, BMI shows a relatively consistent U-shaped association with clinical outcomes, with the best outcomes observed in overweight and mildly obese patients, while low BMI is associated with worse outcomes in all patients, independent of severity of CKD [12]. In contrast, a higher waist circumference was consistently associated with increased mortality [13].

The examination of metabolic syndrome as a risk factor for CKD entails additional complexity. The very definition of metabolic syndrome has eluded full consensus, and even small variations in criteria may affect results in studies of large patient cohorts. Even with efforts towards a more widely accepted definition [14], there may be considerable heterogeneity among patients since only 3 of 5 criteria need to be met, and, thus, different patients might be defined as having metabolic syndrome based on different findings. Additionally, the clustering of several risk factors into one variable may either mask or artificially enhance associations between predictor and outcome and make them more difficult to interpret. Nevertheless, an association between the metabolic syndrome and an increased incidence of CKD appears to be well supported by empirical data [15, 16].

## Main text

The article by Cao et al. in this issue of BMC nephrology is a welcome addition to our knowledge and understanding of this complex issue. It presents the results of a large scale prospective observational cohort study assessing the independent impact of obesity with or without co-existing metabolic syndrome on the risk of new onset CKD [17]. In view of the potentially modifiable nature of obesity and at least some of the other components of metabolic syndrome, information about the relative contribution of each abnormality to the development of CKD has potentially significant clinical implications.

Cao et al. describe a cohort of 6852 Chinese individuals from Changsha, central south China, who were prospectively followed for an average of 54.3 months. The cumulative incidence of new onset CKD (defined as development of either eGFR < 60 mL/min/1.73 m2 or isolated positive proteinuria during the follow up period) in this cohort was recorded [17]. Overweight and obesity was highly prevalent and found in more than 40 % of the study patients. As expected, the incidence of metabolic syndrome significantly and positively correlated with increasing body mass.

Cao et al. showed that the cumulative incidence of CKD increased stepwise with higher BMI category independent of the presence or absence of metabolic syndrome. The hazard ratios for new onset CKD were 1.82 (95 % CI, 1.36-2.42) in overweight and 3.82 (95 % CI, 2.47-6.12) in obese individuals without metabolic syndrome when normal weight individuals without metabolic syndrome were considered as a reference group. More interestingly and similar to individuals without metabolic syndrome, obesity was found to independently increase the cumulative incidences of new onset CKD in those with coexisting metabolic syndrome. The excess risk mediated by metabolic syndrome in the association between BMI (as a continuous variable) and CKD was 26 %. As higher BMI significantly and positively correlated with metabolic syndrome, this finding means that only 26 % of the CKD risk induced by BMI can be explained by associated metabolic syndrome. Not unexpectedly, the risk of CKD rose in stepwise fashion as the number of components of the metabolic syndrome increased, further emphasizing the complex interrelationship between individual components of the syndrome and their simultaneous contributions to the development of CKD.

Thus, this well performed large-scale study shows independent and joint adverse associations between BMI and incident CKD, whereas other metabolic parameters have additive effects on this risk. Although interest in the association between obesity, metabolic health and CKD has been longstanding, several prior large scale studies have missed the critical point of stratification of the analysis by the absence or presence of the metabolic syndrome and, therefore, did not answer the question whether metabolic health modifies this association [6–8]. Thus, Cao et al. should be commended for evaluating this clinically significant point. However, another recently published prospective epidemiological study of 21,840 participants studied associations between obesity, metabolic health, and the risk of end-stage renal disease (ESRD) and yielded different results [18]. This study showed that the metabolic syndrome, but not increased BMI, is a powerful risk factor for ESRD. Moreover, higher BMI was associated with lower risk of ESRD in those without the metabolic syndrome, whereas no association of BMI with ESRD risk was observed in those with the metabolic syndrome. Certainly, these results have added further complexity to our understanding of how obesity, metabolic syndrome and CKD, might be associated. Profound differences in studied populations and methodology can, at least partially, explain the discrepancy between these studies. First, race and age differences were prominent: Cao et al. evaluated young Asian participants, whereas Panwar et al. focused on white and black participants who were significantly older. Indeed, aging is associated with considerable changes in body weight and more importantly, composition [19]. Moreover, the relative risk of adverse outcomes associated with obesity decreases with increasing age, and the BMI value associated with the lowest mortality is higher in older than in younger adults [19]. Though no data evaluating BMI as an independent risk factor for CKD in the elderly exist to date, one would expect that aging can modify the association of BMI with CKD. Additionally, methodological variations can result in different conclusions: Panwar et al. examined the independent associations of BMI and metabolic health with risk of ESRD, while Cao et al. evaluated these associations with risk of early CKD. It is quite possible that increased BMI can be an independent risk factor for early CKD in patients with normal baseline kidney function, but not for ESRD. Moreover, several previous studies have shown that higher BMI may be “protective” in patients with advanced stages of CKD and associated with improved survival [12]. Although the physiological mechanisms underlying this ‘obesity paradox’ are not well understood, poor nutritional status, protein energy wasting and inflammation, which often coexist with lower BMI in advanced CKD, are thought to be major underlying mechanisms responsible for increased mortality in this population [20]. Clearly, the effects of BMI and coexisting metabolic parameters on the risk of development and progression of kidney disease are modified by several important demographic and clinical factors; thus, critical analysis of current literature addressing this complex subject is crucial for clinicians faced with the uncertainty caused by the disparate results of different clinical studies.

The study by Cao et al. [17] was not designed to elucidate mechanisms that may explain the nexus between metabolic syndrome, obesity and risk of CKD. Deleterious effects of intraglomerular hypertension and glomerular hyperthrophy, increased secretion of transforming growth factor beta (TGF-β) leading to glomerulosclerosis, hormonal changes, such as hyperaldosteronism and related pro-inflammatory cytokine secretion, including interleukin 6 (IL-6) and tumor necrosis factor alpha (TNF-α), coexisting lipid disorders and insulin resistance have all been implicated in obesity-driven CKD [21]. There is no doubt that the complex pathophysiology of all three disease states, metabolic syndrome, obesity and risk of CKD, should be an active area of investigation by the clinical and basic scientist alike, especially in light of the potentially modifiable nature of obesity and other metabolic parameters and its impact on patient outcome.

Obesity is associated with the development of multiple medical conditions and comorbid illnesses, including coronary artery disease, stroke, type II diabetes, CKD, premature death and reduced quality of life [5]. It is well established that controlling obesity and the metabolic abnormalities are important to cardiovascular disease prevention. However, very little is known about the effects of weight reduction on CKD development and progression.

Although several animal and human studies point out the positive effects of caloric restriction on albuminuria, blood pressure and glomerular hyperfiltration, the effect of weight reduction on hard renal outcomes is still unknown [21]. Sedentary lifestyle, which is closely linked with obesity and poor metabolic health, has been recently evaluated in conjunction with CKD risk. The extent of a patient’s physical social network, defined as the number of social interactions and personal relationships, education, moderate alcohol intake, and physical activity has been found to be significantly and independently associated with the incidence and progression of early CKD among those with type 2 diabetes [22].

## Conclusions

Modifiable lifestyle and social factors, which often overlap with overweight and obesity, can determine both kidney and cardiac health in people at high cardiovascular and renal risks. Despite the complexity in determining independent associations between BMI, metabolic syndrome and incident CKD, clinicians should ensure that patients with obesity and metabolic syndrome are screened and appropriately managed for early CKD and modifiable lifestyle risk factors, in order to reduce the risk of both renal and cardiovascular morbidity.

## Abbreviations

CKD: Chronic kidney disease
BMI: Body mass index
ESRD: End stage renal disease
GFR: Glomerular filtration rate
WHR: Waist to hip ratio
TGF-β: Transforming growth factor beta
IL-6: Interleukin 6
TNF-α: Tumor necrosis factor alpha
